# Functional genomics in autoimmune diseases

**DOI:** 10.1093/hmg/ddaa097

**Published:** 2020-05-18

**Authors:** James Ding, Antonios Frantzeskos, Gisela Orozco

**Affiliations:** 1 Centre for Genetics and Genomics Versus Arthritis, Division of Musculoskeletal and Dermatological Sciences, School of Biological Sciences, Faculty of Biology, Medicine and Health, The University of Manchester, Manchester M13 9LJ, UK; 2 NIHR Manchester Biomedical Research Centre, Manchester University NHS Foundation Trust, Manchester Academic Health Science Centre, Manchester M13 9WL, UK

## Abstract

Associations between genetic loci and increased susceptibility to autoimmune disease have been well characterized, however, translating this knowledge into mechanistic insight and patient benefit remains a challenge. While improvements in the precision, completeness and accuracy of our genetic understanding of autoimmune diseases will undoubtedly be helpful, meeting this challenge will require two interlinked problems to be addressed: first which of the highly correlated variants at an individual locus is responsible for increased disease risk, and second what are the downstream effects of this variant. Given that the majority of loci are thought to affect non-coding regulatory elements, the second question is often reframed as what are the target gene(s) and pathways affected by causal variants. Currently, these questions are being addressed using a wide variety of novel techniques and datasets. In many cases, these approaches are complementary and it is likely that the most accurate picture will be generated by consolidating information relating to transcription, regulatory activity, chromatin accessibility, chromatin conformation and readouts from functional experiments, such as genome editing and reporter assays. It is clear that it will be necessary to gather this information from disease relevant cell types and conditions and that by doing so our understanding of disease etiology will be improved. This review is focused on the field of autoimmune disease functional genomics with a particular focus on the most exciting and significant research to be published within the last couple of years.

## Introduction

Genome-wide association (GWA) studies have been essential in contributing to our current understanding of autoimmune disease genetics. For example, across three rounds of GWA studies the Wellcome Trust Case Control Consortium gathered data relevant to a host of autoimmune diseases, including ankylosing spondylitis, autoimmune thyroid disease, Crohn’s disease (CD), multiple sclerosis (MS), psoriasis (Ps), rheumatoid arthritis (RA), type 1 diabetes (T1D) and ulcerative colitis (UC) (1–10). Subsequent studies have collected cohorts for additional diseases such as celiac disease (CeD), idiopathic inflammatory myopathies, juvenile idiopathic arthritis, systemic lupus erythematosus and systemic sclerosis (SSc) (11–15). In many instances, original findings have been validated and superseded by ever larger cohorts, imputation to updated reference panels and meta-analyses (16–19).

Significant progress is still being made in cataloging variants for many diseases, especially outside of the European ancestry population, where the size of existing study cohorts is more limited. However, the focus of this review is on bridging the gap between knowledge of genetic associations and an improved understanding of disease pathology, with a particular focus on progress made within the last couple of years. Historically, the significance of a given variant has simply been assigned to the nearest plausible gene, despite the majority of autoimmune variants being located in non-coding regions and there often being no evidence for an impact of a given variant on the assigned gene. Correctly identifying the downstream impact of disease-associated variants is crucial when seeking to translate the findings of genetic studies into patient benefit, e.g. through drug discovery or repositioning.

## Overcoming Linkage Disequilibrium

Because of the non-random inheritance of alleles (linkage-disequilibrium, LD) and sparse coverage of single nucleotide polymorphisms (SNPs) assessed by GWA studies, multiple SNPs may be implicated within a single genetic association. Regions of LD associated with disease-susceptibility can span hundreds of kilobases and may contain multiple genetic elements, such that it is difficult to identify the specific variant which mediates the increased risk associated with a given allele. To overcome this, high-density genotyping can be used to more precisely characterize associations and the impact of LD can be broken down by studying larger and more genetically diverse populations.

The Immunochip Consortium has enabled high-density genotyping of variants associated with many autoimmune diseases (20,21–32), and this has continued to yield valuable insights into autoimmune disease genetics. A meta-analysis of Immunochip data from a total of 37 000 patients with either CeD, RA, SSc or T1D and 22 000 controls has better characterized the high degree of genetic overlap between these diseases, identifying 38 variants with some degree of pleiotropy (33). These variants were enriched for permissive epigenetic marks in T cells and especially T-helper 17 and regulatory T cells, highlighting the potential for genetic information in informing a model of disease pathogenesis. Such studies demonstrate both the complexity of autoimmune genetics and the potential value of additional genetic analyses.

Epigenetic marks or other such functional annotation data can be used to infer the likelihood of SNPs being causal. A recent study fine-mapped RA and T1D loci and additionally collated publically available enhancer and promoter annotations, as well as expression quantitative trait loci (eQTL) data, H3K4me3 occupancy (indicative of regulatory regions), chromatin accessibility (based on DNAse I hypersensitivity) and transcription factor binding site occupancy/motif disruption (34). This allowed them to infer which SNPs within a given credible SNP set may be most likely to confer risk. Potentially causal variants were identified at a number of loci, with functional assays confirming allele-specific effects on protein binding and enhancer activity for variants proximal to *CD28*/*CTLA4*, *MEG3* and *TNFAIP3*.

## Identifying the Right Cell Type

It is clear that both the quality of the functional data and relevance of the sample from which it originates are fundamental to its utility. The latest release of eQTL data from the Genotype-Tissue Expression project (70–361 samples for 44 different tissues) is estimated to explain ~55% of the variant-based heritability for UC (35). It is likely that this high percentage may be partly down to the inclusion of several tissue types of high relevance to UC, with the highest heritability enrichment being shown in transverse colon, whole blood, spleen, small intestine and sigmoid colon. Increasing this percentage and matching it for other autoimmune diseases may require increasing sample sizes and alternative sample types.

The most relevant sample type may well be disease relevant cells isolated from patients and to this end, a recent study performed RNA-sequencing and profiled H3K27ac and H3K4me1 (indicative of active or primed enhancers) in T-helper cells and regulatory T cells from six T1D patients and five age-matched healthy controls (36). The authors found a higher degree of enrichment for T1D variants among enhancers identified in their study than in alternative annotations, although this was not significantly different between T1D-specific enhancers and healthy control-specific enhancers. Despite very low numbers, variants found within two of the enhancers identified, intronic to *CD69* and *UBASH3A*, were found to be associated with changes in histone marks and differential gene expression of *CD69* and *UBASH3A*. Similarly, an alternative study using T-helper cells and B cells from 344 untreated RA patients, discovered eQTLs for a total of 14 genes at 10 RA loci, roughly half of which were specific to either cell type (37).

Advances in single-cell technologies have contributed enormously to the generation of data from ever more precisely defined cell types. For example, single-cell RNA sequencing (scRNA-seq), mass cytometry and flow cytometry on T cells, B cells, monocytes and fibroblasts from RA and osteoarthritis synovial tissue samples identified and characterized subpopulations that are expanded in RA synovium and may mediate RA pathogenesis (38). The feasibility of identifying cell-type specific eQTLs from scRNA-seq data has been demonstrated in a separate study using ~25 000 peripheral blood mononuclear cells from 45 donors (39).

## Making Contact

Understanding the 3D conformation of the genome can help to provide a mechanistic link between local changes in chromatin and an effect on the expression of protein-coding genes. It can even provide a quantitative association between variant containing loci and genes when eQTL, or other QTL data are absent, thereby identifying downstream effects of potentially causal variants. Data from perturbation of thousands of enhancers and fluorescence-based detection of transcription have recently informed a mathematical model that stipulates that an enhancer’s function is equivalent to the product of interaction frequency and permissive chromatin modifications (40).

Sequencing-based chromatin conformation capture techniques, such as Hi-C, enable chromatin interactions to be mapped genome wide. Hi-C data provide a heatmap of interaction frequency for all genomic intervals that reveal multiple layers of organization, with chromatin being segregated into domains where interactions occur more frequently within than outwith the region. The resolution of such maps is limited by the restriction enzymes used during library generation and by sequencing depth, but it is clear that eQTLs aggregate within the highest order domains: topologically associated domains (41), which are frequently associated with CTCF sites (42).

While the higher order features of chromatin architecture are considered to be reasonably stable across cell types and individuals, at individual loci and higher resolution, there is more variability. In keeping with this, a recent study performed Hi-C on lymphoblastoid cell lines from 20 individuals, identifying thousands of regions across the genome where chromatin conformation varies and is accompanied by changes in histone modifications, transcription factor binding and gene expression (43). Furthermore, it was possible to associate common variants with various quantifiable chromatin conformation phenotypes, including directionality imbalance, insulation and interaction frequency. Insulation-QTLs were enriched for nominally associated UC and inflammatory bowel disease (IBD) variants, whereas interaction frequency-QTLs showed an enrichment that fell just below the level of significance. It will be interesting to see how future studies using more disease relevant cell types, larger sample sizes and better matched GWA studies and Hi-C populations build on this.

To improve sequencing depth, it is possible to use oligonucleotide baits to ‘capture’ a defined proportion of a Hi-C library before sequencing (CHi-C). Baits can span features of interest, such as promoters (promoter CHi-C) (44) or autoimmune loci (45,46). CHi-C data mapping interactions at JIA, RA and psoriatic arthritis (PsA) loci in T-helper and B-cell lines have recently been combined with chromatin annotations from relevant cell types to generate a list of genes whose promoter is found to interact with an enhancer that harbors disease susceptibility (47). These genes are significantly enriched in disease relevant pathways and include targets both for existing therapies and drugs which could potentially be repositioned and tested for efficacy in these diseases.

HiChIP involves performing chromatin immunoprecipitation (ChIP) on a Hi-C library, using a protein of interest such as H3K27ac, such that the resulting data contain information regarding protein occupancy and interaction frequency (48). CHi-C and H3K27ac HiChIP have recently been applied to keratinocyte and skin resident cytotoxic T-cell lines, leading to the validation of existing potentially causal genes for Ps, as well as the identification of novel candidates (49). A more extensive analysis of this data, including other publicly available Hi-C, CHi-C and HiChIP data have identified relevant genes for Ps, PsA and SSc and highlighted the enhanced tissue-specificity of H3K27ac HiChIP data (50).

As an example of how these techniques can be combined, a recent study compared chromatin accessibility (assay for transposase-accessible chromatin [ATAC]), CTCF occupancy and chromatin conformation in thymocytes from a T1D mouse model (NOD) and diabetes-resistant mice (51). The authors identified a higher number of chromatin interactions in the *Idd* T1D locus in NOD mice and analyzed this and other hyper-connected diabetes-specific 3D niches in depth, using fluorescence-microscopy and HiChIP.

## Experimental Characterization of Individual Loci

**Figure 1:**
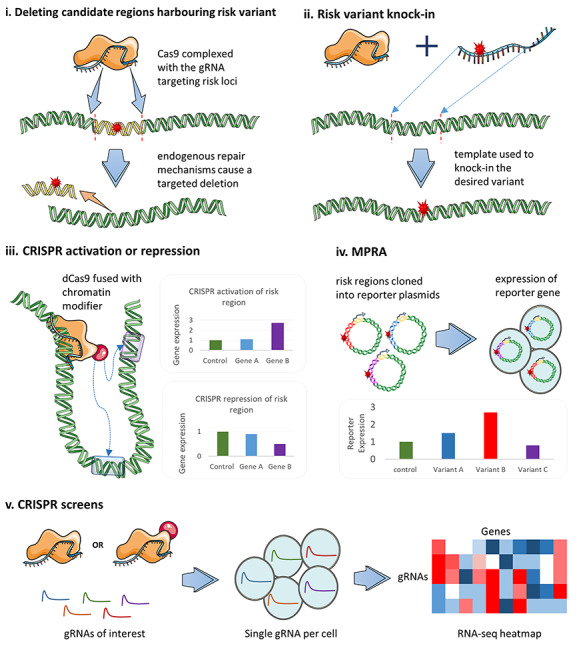
The functional role of individual risk variants are validated experimentally using a range of CRISPR/Cas9 technologies and in a high-throughput manner by MPRA and CRISPR screens. Examples include: (**A**) Deletion of disease-associated risk loci by targeting Cas9 to either side. (**B**) Knock-in of risk variants by introducing a repair template containing a risk variant and homology to the cut site. (**C**) Artificial activation or repression of loci using dCas9 fused to a chromatin modifier. (**D**) MPRAs where candidate regions and variants are cloned into reporter plasmids. (**E**) CRISPR screens using scRNA-seq or phenotypic readouts for simultaneously screening the effect of many gRNAs targeting regions of interest. Cas9, CRISPR-associated protein 9; CRISPR, clustered regularly interspaced palindromic repeats; dCas9, catalytically dead Cas9; gRNA, guide RNA; MPRA, massively parallel reporter assay.

To investigate or validate mechanisms by which disease susceptibility mechanisms are mediated various experimental approaches are used, including reporter assays and genomic perturbation (Fig. 1).

Reporter (e.g. luciferase) assays are used to assess the regulatory impact of candidate DNA regions, such as those harboring candidate SNPs. This can be performed in a high-throughput manner as part of massively parallel reporter assays (MPRAs) and has recently been optimized for use in primary T-helper cells (52). This technique was applied to 14 autoimmune loci, in rested and stimulated primary T-helper cells, identifying SNPs which had the greatest impact on regulatory activity. For example, out of 44 candidate SNPs within the 6p23 IBD and MS risk locus, rs34421390 was identified as most likely to affect regulatory activity. Similarly, rs6927172 was identified with a significant regulatory role out of hundreds of non-coding SNPs at the 6q23 CD, CeD, RA, SLE, T1D and UC risk locus.

A major drawback to MPRAs is that each SNP is examined outside of its native environment. It is possible to overcome this using the latest genomic editing tool, clustered regularly interspaced palindromic repeats (CRISPR)/CRISPR-associated protein 9 (Cas9). In the aforementioned study, Cas9 was targeted to multiple locations within close proximity of rs6927172 in primary T-helper cells, giving rise to a heterogeneous combination of deletions and other edits. Edited cells had reduced *TNFAIP3* expression, with no change in five other neighboring genes (52). A similar approach was applied separately in HEK293 cells, generating clonally derived lines with specific deletions of 11/12 bp including rs6927172. The authors also found that these deletions lead to reduced *TNFAIP3* expression, but in contrast to the results from primary T-helper cells, they also saw a reduction in *IL20RA* and *OLIG3* expression (53).

While there are limited examples of CRISPR screens being applied specifically to autoimmune disease functional genomics, it is clear from related studies that these techniques are highly relevant. For example, a conventional ‘knockout’ CRISPR screen coupled with ChIP-sequencing and ATAC-sequencing data has identified new genes influencing the differentiation of naïve T-helper cells into type 2 T-helper cells using murine primary T cells (54). CRISPR/Cas9 screens have also been developed for use in primary human T cells, identifying genes regulating T-cell proliferation following stimulation (55).

In addition to the generation of deletions, or specific edits, CRISPR/Cas9 can be used to modify chromatin activity (using catalytically inactivate dCas9 fused to chromatin modifiers). A comparison of various methods used for characterizing autoimmune variants was recently published, focused on multiple autoimmune loci found within the 6q23 region (56). The authors performed a number of observational techniques, assaying chromatin accessibility (using both DNAse I and ATAC) and activity (using H3K27ac), as well as assessing the impact of variants through two MPRAs (differing based on the method of reporter plasmid delivery) and through two CRISPR screens (both activating and repressing chromatin), all in monocyte, B-cell and T-helper cell lines. The authors found that many of their assays gave uninformative or contradictory information with repression of chromatin being the most informative. It should be noted, however, that chromatin repression was only performed when variants were in or near regions of active chromatin (6.3% of variants included) and that while many chromatin modifying CRISPR-screens use scRNA-seq as a readout the authors used their own method of fluorescence-based detection of transcription.

## Conclusion

Using a combination of genetic, transcriptomic, epigenetic and chromatin conformation data, it is possible to refine associations with autoimmune disease susceptibility to identify potentially causal variants and genes. The techniques required to generate these data are constantly being improved, such that they are becoming accessible for a wider variety of samples, in greater numbers. Once identified, the effects of potentially causal variants can be investigated or validated through experimentation, often involving reporter assays or genome editing technologies.

The potential benefits of these approaches have been demonstrated by many studies, e.g. by generating a priority index of potential drug targets for 30 immune traits (57). Targets were identified using genetic, eQTL and chromatin conformation data and subsequently validated using a CRISPR screen. As in this example, the consolidation of data from a variety of cell types, techniques and sources is likely to be crucial. The value of this has been demonstrated using a novel method for annotating cell state-specific regulatory elements, trained using over 500 chromatin and sequence annotations. This annotation was able to capture a greater proportion of heritability to RA in T-helper cells than is possible using either histone marks or expressed genes alone (58).

By implementing these techniques in a sufficient number of relevant samples and combining the data, we stand to improve our understanding of autoimmune disease and translate genetic findings into patient benefit.
